# Retraction: LncRNA MALAT1up-regulates VEGF-A and ANGPT2 to promote angiogenesis in brain microvascular endothelial cells against oxygen-glucose deprivation via targeting miR-145

**DOI:** 10.1042/BSR-20180226_RET

**Published:** 2020-07-21

**Keywords:** Stroke, BMECs, lncRNA-MALAT1

This Retraction follows an Expression of Concern relating to this article previously published by Portland Press.

This article is being retracted from *Bioscience Reports* at the request of the Editor-in-Chief and the Editorial Board following receipt of a notification from several readers, alerting the Editorial Board to multiple duplications in Figures 2D, 2E, 4D and 4E.

The authors have been contacted in regards to the retraction, and have not responded to the Journal's queries and the concerns raised. Given the extent of the issues raised, the Editorial Board stand by the decision to retract the article.

